# Learning styles and approaches to learning among medical undergraduates and postgraduates

**DOI:** 10.1186/1472-6920-13-42

**Published:** 2013-03-25

**Authors:** Lasitha Samarakoon, Tharanga Fernando, Chaturaka Rodrigo

**Affiliations:** 1 National Hospital, Colombo, Sri Lanka; 2 Department of Clinical Medicine, Faculty of Medicine, University of Colombo, Colombo, Sri Lanka

**Keywords:** Learning methods, Medicine, Post graduate, Undergraduate curriculum evaluation

## Abstract

**Background:**

The challenge of imparting a large amount of knowledge within a limited time period in a way it is retained, remembered and effectively interpreted by a student is considerable. This has resulted in crucial changes in the field of medical education, with a shift from didactic teacher centered and subject based teaching to the use of interactive, problem based, student centered learning. This study tested the hypothesis that learning styles (visual, auditory, read/write and kinesthetic) and approaches to learning (deep, strategic and superficial) differ among first and final year undergraduate medical students, and postgraduates medical trainees.

**Methods:**

We used self administered VARK and ASSIST questionnaires to assess the differences in learning styles and approaches to learning among medical undergraduates of the University of Colombo and postgraduate trainees of the Postgraduate Institute of Medicine, Colombo.

**Results:**

A total of 147 participated: 73 (49.7%) first year students, 40 (27.2%) final year students and 34(23.1%) postgraduate students. The majority (69.9%) of first year students had multimodal learning styles. Among final year students, the majority (67.5%) had multimodal learning styles, and among postgraduates, the majority were unimodal (52.9%) learners.

Among all three groups, the predominant approach to learning was strategic. Postgraduates had significant higher mean scores for deep and strategic approaches than first years or final years (p < 0.05). Mean scores for the superficial approach did not differ significantly between groups.

**Conclusions:**

The learning approaches suggest a positive shift towards deep and strategic learning in postgraduate students. However a similar difference was not observed in undergraduate students from first year to final year, suggesting that their curriculum may not have influenced learning methodology over a five year period.

## Background

Teaching medicine is an ever-evolving process which requires that both students and teachers continuously update themselves. The challenge of imparting a large amount of knowledge within a limited time period in a way it is retained, remembered and effectively interpreted by a student is considerable. This has resulted in crucial changes in the field of medical education, with a shift from didactic teacher-centered and subject-based teaching to the use of interactive, problem-based, student-centered learning. Most medical school curricula have adopted new methods of teaching and learning to varying degrees [1]. It has been argued that knowledge of learning styles can be useful to both teachers and students, in that teachers can tailor pedagogy to correlate with the learning styles of students [2,3]. Similarly, students with knowledge of their learning styles could be empowered to identify and use the techniques of learning best suited to their individual styles, resulting in greater educational satisfaction.

Dunn et al. [4] defined the term ‘learning style’ as different and unique ways used by individuals as they prepare to learn and recall information. Educational theory suggests that clinical experience and success at examinations bears a relationship to learning styles. School performance has been shown to correlate poorly with students’ performance in the university [5], possibly because university education requires more deep learning and analytical thinking compared to simple factual recall required for advanced level or equivalent school examinations. Nonetheless, some students seem to cope with the challenge of adopting deep learning better than others, and perform well at undergraduate and postgraduate levels.

Learning styles have been shown to vary widely among students; the VARK learning styles inventory measures four sensory modalities used for learning, namely Visual, Aural, Read/Write and Kinesthetic [6]. According to individual preference to learning style, learners can be classified as unimodal if they show predominantly one learning preference or multimodal if preference is shared between 2 or more learning styles.

Students learn by relying on understanding, by relying on rote memorization and reproducing memorized information, or by a combination of these methods to varying degrees [7]. Three different approaches to learning have been identified, viz., deep approach (DA), surface apathetic approach (SAA) and strategic approach (SA) [8]. DA is an organized approach where emphasis is placed on understanding concepts and relating ideas, and is considered the preferred style of learning in university education. SAA, on the other hand, is syllabus bound superficial learning with emphasis on route memorization. SA students use either deep or superficial learning as appropriate for a particular topic, with the aim of achieving highest possible grades. This type of learning is characterized by alertness to assessment and monitoring, and results in fragmented understanding of subject matter, with poor integration across topics [8]. While SAA is more likely to result in failure in university final examinations, both DA and SA are more likely to result in success [9].

Thus various questionnaires have been developed over time to indicate students’ overall approaches to learning and their perceptions of the teaching-learning environments, as well as related aspects of students’ attitudes and experience. We have utilized one such questionnaire, developed by the Enhancing Teaching-Learning Environments (ETL) project in the United Kingdom - The Approaches to Study Skills Inventory for Students (ASSIST).

ASSIST is a useful instrument for providing accessible learning related information which students can reflect upon [6]. The ASSIST questionnaire asks students about their study habits, and classifies responses according to the three approaches to learning, i.e., deep, strategic and surface/apathetic.

There are minimal published data on learning styles and approaches among undergraduates in Sri Lankan medical schools. Secondary education in schools is largely didactic lecture based, encouraging students towards auditory and read/write learning styles. The General Certificate in Education - Advanced Level examination is a norm-referenced competitive examination which determines entry to the universities. The medical school of the University of Colombo, Sri Lanka, has an integrated modular curriculum with a significant emphasis on problem based learning. The curriculum utilizes many diverse methods of learning, and assessments include a significant continuous assessment component. The final assessment is a criterion-referenced qualifying examination. We thus hypothesized that there would be significant differences in learning styles and learning approaches seen between first year and final year students; i.e., first year students would be expected to favour auditory and read/write learning styles and strategic learning, while final year students would be expected to switch to multimodal learning styles with greater emphasis on deep learning. Furthermore, postgraduate training is largely based on on-the-job training, with very little didactic learning. Thus we also hypothesized that postgraduate trainees would favour kinesthetic learning styles and deep learning. This study was conducted to determine whether such differences existed between these three cohorts. Identifying such differences in learning styles and approaches could potentially be used to tailor these curricula to encourage diverse learning styles, and to encourage deep learning rather than strategic and superficial learning.

## Methods

This study analyses the learning styles and approaches to learning in cohorts of undergraduate students in first (preclinical) year and final (clinical) year in the University of Colombo as well as postgraduate trainees of the Postgraduate Institute of Medicine, University of Colombo, Sri Lanka. The study was conducted in 2012. The undergraduate curriculum in the University of Colombo is an integrated modular curriculum, with five main streams running through the study course; the introductory basic sciences stream, the applied sciences stream, the behavioural stream, the community stream and the clinical stream, with a combination of continuous assessments and end of course assessments determining successful performance. The postgraduate study program is based on an apprenticeship model with on the job training, work-place based assessments, self-study and professional exit clinical examinations.

The study instruments used were validated ASSIST and VARK questionnaires. In the case of undergraduates, the questionnaires were distributed to students in the first year and final year during lectures and practical sessions. Alternate students in the attendance registers of first and final years were chosen, and consenting students were invited to participate in the study. Postgraduate trainees stationed in the National Hospital, Colombo (the affiliated teaching hospital) were approached individually by two investigators during work hours and consenting individuals were invited to participate in the study. Care was taken to avoid replication by requesting the participants to confirm that they had not completed the questionnaire at an earlier session. In all instances, participants were briefed as to the objectives of the study, and confidentiality of responses was ensured by maintaining anonymity of responders.

English language versions of both questionnaires were self administered. Each response was scored according to protocols developed by the developers. In the VARK questionnaire, we first calculated subscale scores according to protocol, and then determined preferred learning approach and unimodality or multimodality according to sub scale scores. Similarly in the ASSIST questionnaire, subscale scores were calculated for each approach individually, and the predominant learning approach was calculated using the subscale scores according to protocol. All data were entered in to a SPSS database. Data was analyzed using SPSS v15, and the Student’s T-test was used for statistical comparisons. Ethics clearance for the project was obtained from the Ethics Review Committee of the Faculty of Medicine, University of Colombo.

## Results

### Demographic data

A total of 147 students participated in the study: 73 (49.7%) first year students, 40 (27.2%) final year students and 34 (23.1%) postgraduate students. Respondent rates were 98.6%, 88% and 94.1% respectively. The mean age of participants was 20.9 (standard deviation [SD] ± 1.08) years in the preclinical group, 26.2 (SD ± 1.11) years in the clinical group and 32.9 (SD ± 2.66) years in the postgraduate group. The male:female ratio of participants was 1.1:1 with 77 (52.4%) male participants and 70 (47.6%) females.

### Learning styles

**Figure 1:**
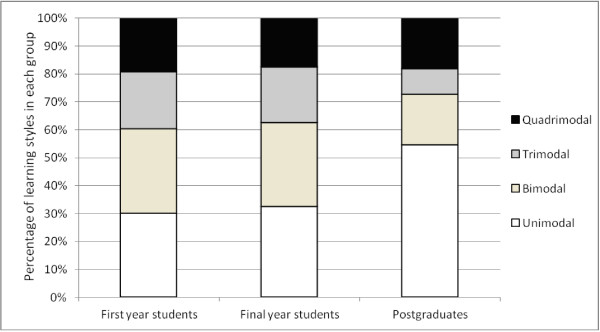
Graphic representation of the VARK inventory results for learning styles among preclinical, clinical and postgraduate groups.

The majority (69.9%) of first year students had multimodal learning styles (Figure 1). Among the unimodal learners (30.1%), the clear majority were auditory learners (50%). Among multimodal learners, 30.1% were bimodal learners with auditory-reading (50%) and auditory-kinesthetic (31.8%) types predominating. Similarly among final year students, the majority (67.5%) preferred the multimodal approach (Figure 1) with (30%) having bimodal type. Just 32.5% were unimodal learners with the 38.5% having kinesthetic type. The proportion of unimodal learners was statistically similar between pre-clinical and clinical students (p = 0.79). Among postgraduates, the majority were unimodal (52.9%) learners with 33.4% having kinesthetic type. Postgraduates were statistically more likely to be unimodal learners compared to the undergraduates (p = 0.019).

### Learning approaches

Among all three groups, the predominant approach to learning was SA. (Table 1) Comparing the different groups, postgraduates had significantly higher mean scores for DA and SA than first years or final years (p = 0.0128 and p = 0.0338 respectively for first years, p = 0.0058 and p = 0.0064 respectively for final years). Mean scores for the SAA did not differ significantly between groups. Mean scores for all three approaches did not differ significantly between first and final years (Table 1).

**Table 1:** Comparison of mean ASSIST scores among preclinical, clinical and postgraduate groups

	**Pre clinical vs clinical**	**Pre clinical vs post graduates**	**Clinical vs post graduates**
Deep approach [DA]	57.11 Vs 55.92	57.11 Vs 61.15	55.92 Vs 61.15
	p = 0.4558	p = 0.0128*	p = 0.0058*
Surface Apathetic approach [SAA]	50.93 Vs 66.28	50.93 Vs 50.06	66.28 Vs 50.06
	p = 0.6356	p = 0.6858	p = 0.9450
Strategic approach [SA]	68.99 Vs 66.28	68.99 Vs 73.91	66.28 Vs 73.91
	p = 0.2644	p = 0.0338*	p = 0.0064*

Mean scores for the learning approaches were individually calculated for each of the three groups and compared using Student’s T-test; *indicates statistically significant difference ay p < 0.05.

## Discussion

Our study revealed several interesting differences among undergraduates and post graduates with regards to learning styles and approaches. To begin with the response rates in our study was altogether quite high. Even so when compared with pre-clinical and postgraduate groups response rate was somewhat lower in the clinical group. Post graduates were individually approached by the investigators, and that may explain the high response rate in that group. Our study showed that the majority of undergraduate students had multimodal learning styles, with auditory learning being a predominant component. In a similar study conducted in USA, the majority (63.8%) had multimodal learning styles with only 36.1% having a unimodal learning preference; however auditory learners were only a small minority (4.8%) [10]. Another study in Turkey showed similar results with the multimodal approach being the predominant style (63.9%), with only 3.2% being auditory learners [11]. Both these studies demonstrated a clear predominance of kinesthetic learners (18.1% and 23.3% respectively) among unimodal learners. A similar study conducted in Australia among first year nursing students demonstrated a predominance of the kinesthetic style of learning [12]. The differences observed in our students may be attributable to the pre-university education system in the country, where students traditionally follow didactic lectures in schools. Pre-university education is often supplemented with private tuition classes; these could be either small group classes or larger lecture based classes. They primarily promote strategic learning, since satisfactory performance at the university entrance examinations reinforces the credibility and popularity of the tutors conducting these classes.

Although multimodal preference with auditory learning is predominant among pre-clinical undergraduates, learning styles do seem to change as they move up the ladder of medical education. Despite the majority remaining multimodal learners, a shift is seen to occur from predominantly auditory to predominantly kinesthetic learning from first to final years. Unfortunately, despite there being an integrated modular curriculum with an emphasis on modern learning and assessment methods such as problem-based learning, small group discussions and continuous assessments, the fundamental learning styles do not appear to have changed significantly over the five years of undergraduate medical education, as evidenced by minimal change in the proportion of multimodal learners (69.9% among first years to 68.5% among final years).

A more significant step up occurs after actually starting to work as doctors. Kinesthetic learning predominates among postgraduates, and here a more dramatic shift is seen towards unimodal learning. The reason(s) for this shift is obscure; exposure to clinicals in the practical setting where the focus changes from didactic learning to practicality, as well as reduced amount of lecture time and encouragement by trainers to develop self-learning skills are probable reasons. The mean age of postgraduate students was 7 years above the preclinical first years, and this fact may also play a role in the shift toward a more kinaesthetic type of learning observed among postgraduates. Institutional differences in teaching methodology may also explain such learning behaviours. For example post graduates, are attached to PGIM where the training programme is based upon self-learning apprenticeship model with more emphasis on in service training rather than didactic lecture based learning given to undergraduates.

Encouraging results have been obtained from a study among Jordanian nursing students, where Problem-Based-Learning (PBL) was introduced as an improvement to the conventional curriculum. A significant improvement in learning style, as evidenced by an increase in mean VARK score and percentage increase of multimodal learners in pre and post tests, was seen after introduction of PBLs in a Jordanian study [13]. Thus, it is likely that activities which promote active learning such as discussions, debates and role playing may enhance the learning experience of students. Our curriculum has components of these activities, but the desired effect does not seem to have been achieved. Because of the large numbers of undergraduates, (approximately 200 per batch) most PBLs and Small Group Discussions (SGDs) often comprise larger groups, of up to 20 students; larger groups discourage active learning, and the weaker students are more likely to fall back towards auditory learning. This is a difficult problem to remedy, given the financial and logistic constraints in universities in developing countries like Sri Lanka. Strategies to improve the quality of PBLs and SGDs, such as training tutors are recommended.

Strategic learning was the predominant learning approach in all three groups, i.e., pre-clinical, clinical and postgraduates. However, post graduates had significantly higher scores for deep approach and strategic approach than undergraduates (p < 0.05), although scores did not differ significantly for any approach between pre clinical and clinical undergraduates. Although an increase in the superficial approach to learning was noted in undergraduates during the progression of time in an Australian study,[14] such a trend was not observed in another study conducted in Indonesia [15]. Mean scores for the SAA remained constant among undergraduates in our study. In another recent study [16], it was noted that pre-clinical students and postgraduate trainees had the highest mean for DA, while clinical students had the highest mean for SAA and pre-clinical students for SA. This is in contrast to our study where postgraduate students had highest mean scores for the DA and SA, while the highest mean scores for SAA were similar among the pre-clinical students and clinical students. In our study, out of the three groups, the lowest scores for the deep approach was noted among clinical students. Similar results were seen in a previous Australian study [14] but contradictory results were seen in a study conducted in Indonesia [15]. Although our study confirms that there are significant differences in learning approaches among medical undergraduates and postgraduates, this does not seem to be the norm in non-medical fields. Having come thus far, how can we explain such differences among medical undergraduates and postgraduates? Reasons for such learning styles and approaches may be multifactorial. The need to compete for grades was identified as a factor promoting superficial learning among new medical undergraduates in a study conducted in United States of America [17]. Higher workload with increasingly tight course schedules may promote superficial and assessment oriented strategic learning among undergraduates in preference to the deep approach. Predictability of assignments may be another factor encouraging strategic approach to learning. The evidence for any one approach to be more successful in medical school performance is controversial at best, some studies showing no relationship between examination success and a specific learning approach [18], and others showing that DA and SAA result in better examination performance [19]. Since the postgraduate group had significantly higher mean scores for the DA and SA, our study would also support the fact that DA and SA may be associated with better performance at post graduate entrance examinations.

### Limitations

Our study had several limitations. Firstly, there is little evidence that learning styles really do make a difference to learning [20]. Nonetheless, knowledge of learning styles and approaches can be used to tailor curricula to suit the majority of students. Secondly, our study was cross sectional rather than longitudinal. Thus we were only able to describe differences between the three cohorts studied, and no firm conclusions can be drawn regarding changes in learning styles and approaches over time.

## Conclusions

Learning styles and learning approaches differ among medical undergraduates as well as undergraduates and post graduates. The learning approach suggested a positive shift towards deep and strategic learning in postgraduate students. However a similar difference was not observed in undergraduate students during their transition from first year to final year. Differences in the learning styles and learning approaches have important implications in development of effective medical curricula in both undergraduate and post graduates.

## Abbreviations

DA: Deep approach; SAA: Surface apathetic approach; SA: Strategic approach.

## Competing interests

The authors declare that they have no competing interests.

## Authors’ contributions

LS conceptualized and planned the project under the guidance of CR and SR. LS and TF gathered and analyzed data. LS prepared the initial manuscript. CR and SR made critical revisions to the manuscript. All authors read and approved the final manuscript.

## Pre-publication history

The pre-publication history for this paper can be accessed here:

http://www.biomedcentral.com/1472-6920/13/42/prepub
